# Anti‐Melanoma Differentiation‐Associated Gene 5 Antibody‐Positive Interstitial Lung Disease, Induced by Enfortumab Vedotin Plus Pembrolizumab for Advanced Urothelial Carcinoma

**DOI:** 10.1002/iju5.70148

**Published:** 2026-02-05

**Authors:** Akira Saito, Hiroki Ishihara, Hanae Kondo, Toshio Takagi, Yasunobu Hashimoto

**Affiliations:** ^1^ Department of Urology Saiseikai Kawaguchi General Hospital Saitama Japan; ^2^ Department of Urology Tokyo Women's Medical University Tokyo Japan

**Keywords:** anti‐melanoma differentiation‐associated gene 5 antibodies, bladder cancer, dermatomyositis, enfortumab vedotin plus pembrolizumab, immune‐related adverse events

## Abstract

**Introduction:**

Anti‐melanoma differentiation‐associated gene 5 antibody‐positive interstitial lung disease is a rare but fatal adverse event following immune checkpoint inhibitor treatment for cancers. However, such cases have not been previously reported in patients with urothelial carcinoma.

**Case Presentation:**

A 79‐year‐old Japanese woman with advanced unresectable bladder cancer was treated with first‐line enfortumab vedotin plus pembrolizumab. During the second treatment cycle, interstitial lung disease developed, and the patient immediately received steroid pulse therapy. As interstitial lung disease rapidly progressed after the initiation of steroid pulse therapy, immunosuppressive treatment with cyclophosphamide and tacrolimus was added. However, interstitial lung disease did not improve, and the patient died on day 54 after initiating treatment. Detailed antibody examination revealed positive anti‐melanoma differentiation‐associated gene 5 antibodies.

**Conclusion:**

We report the first case of anti‐melanoma differentiation‐associated gene 5 antibody‐positive interstitial lung disease induced by enfortumab vedotin plus pembrolizumab in a patient with advanced urothelial carcinoma.

## Keynote Message

We report the first known patient with urothelial carcinoma who developed rapidly progressive interstitial lung disease (ILD), with anti‐melanoma differentiation‐associated gene 5 (MDA5) antibody positivity, induced by enfortumab vedotin plus pembrolizumab. This case serves as an important clinical reference for managing adverse events caused by immune checkpoint inhibitors. Our findings emphasize how essential early screening for anti‐MDA5 antibodies is in patients with rapidly progressive ILD.

## 1 Introduction

Immune checkpoint inhibitors (ICIs) are commonly used as a standard of care for advanced cancers, including urothelial carcinoma (UC). In a recent pivotal clinical trial, the EV‐302 trial, enfortumab vedotin plus pembrolizumab (EV + P) significantly improved overall survival when compared with platinum‐based chemotherapy in patients with advanced UC [1]. Based on this evidence, EV + P is now recognized as a first‐line systemic therapy for advanced UC in current guidelines [2].

ICIs can induce various types of adverse events (AEs), including immune‐related AEs (irAEs) [3]. As some AEs can be fatal [4], prompt and appropriate treatments are required.

Herein, we report on a patient with unresectable advanced bladder cancer who developed rapidly progressive interstitial lung disease (ILD), harboring an anti‐melanoma differentiation‐associated gene 5 (anti‐MDA5) antibody following first‐line treatment with EV + P. To our best knowledge, this is the first report on the development of anti‐MDA5 antibody‐positive ILD after ICI treatment for UC.

## 2 Case Presentation

**FIGURE 1:**
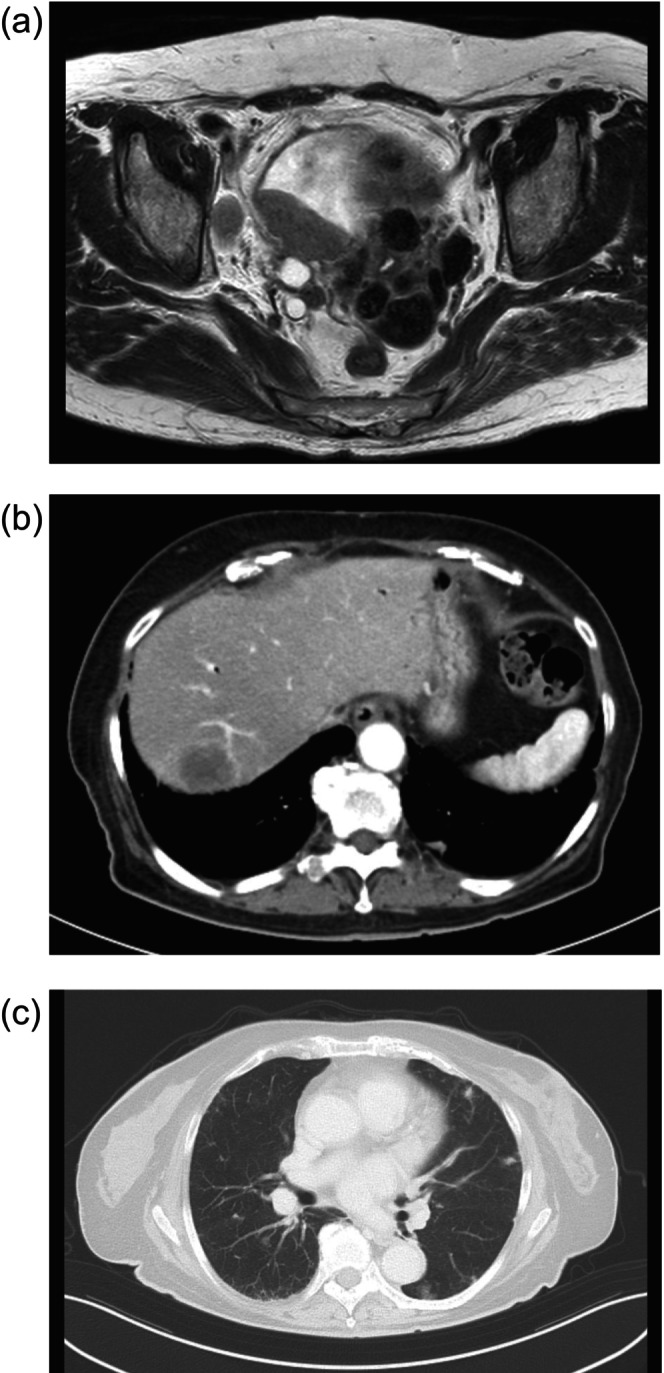
Computed tomography (CT) scan imaging at the time of diagnosis. The patient had massive bladder cancer with multiple lymph nodes, lung, and liver metastases. (a) In the pelvis. (b) In the liver. (c) In the lungs.

A 79‐year‐old Japanese female was referred to our hospital with macroscopic hematuria. Cystoscopy revealed a 3‐cm basal nonpapillary tumor extending from the right ureteral orifice to the bladder neck. Contrast‐enhanced magnetic resonance imaging revealed extramural invasion of the bladder tumor and lymph node metastasis (Figure 1a). We performed a transurethral resection of the bladder tumor, and the patient was pathologically diagnosed with high‐grade pT2 UC. Contrast computed tomography scan further revealed multiple lymph node, lung, and liver metastases (Figure 1b,c). Based on these findings, the patient had unresectable metastatic bladder cancer (cT2N1M1), and EV + P was administered as first‐line systemic therapy [1, 5]. The EV + P dose was reduced to 80% owing to her impaired general condition (Karnofsky Performance Status score of 70), older age, and liver dysfunction.

**FIGURE 2:**
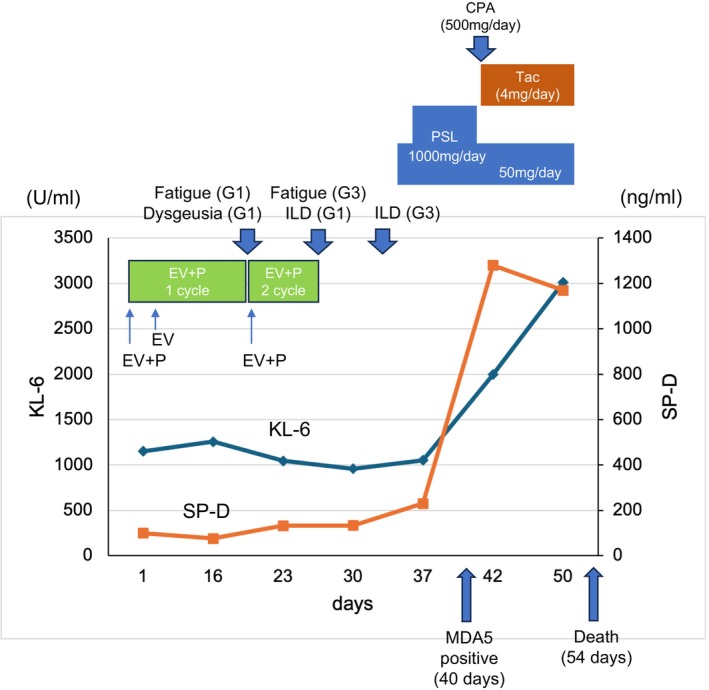
Timeline showing clinical course. CPA, cyclophosphamide; EV + P, enfortumab vedotin plus pembrolizumab; ILD, interstitial lung disease; PSL, prednisolone; Tac., tacrolimus.

**FIGURE 3:**
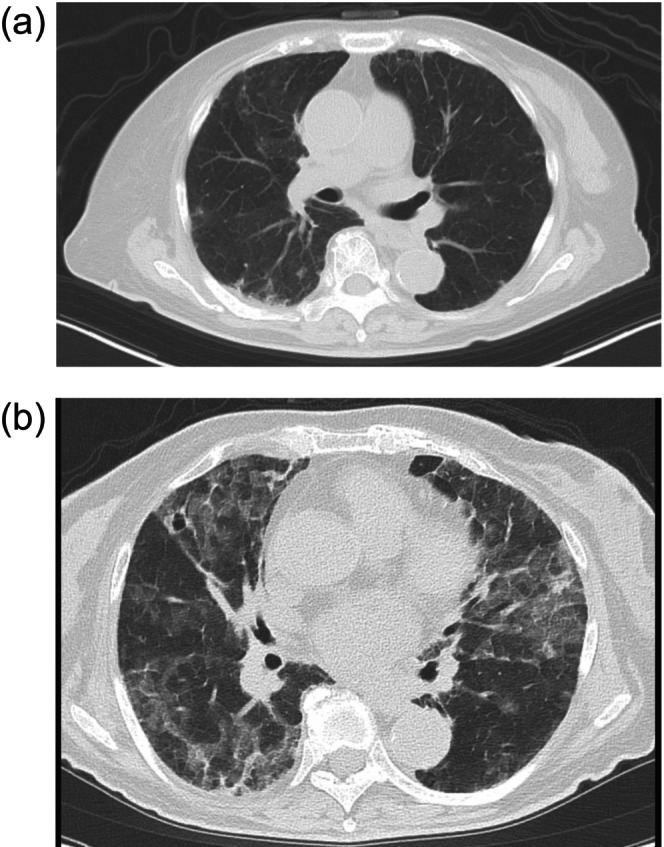
Timeline of CT scan imaging of interstitial lung disease (ILD). On day 8 of the second cycle of enfortumab vedotin plus pembrolizumab, ILD developed to grade 1. One week later, ILD worsened to grade 3. (a) Development of ILD. (b) Exacerbation of ILD. ILD, interstitial lung disease.

Fatigue and dysgeusia of Common Terminology Criteria for Adverse Events grade 1 were observed during the first cycle of EV + P treatment (Figure 2) [6]. On day 8 of the second cycle, fatigue worsened (grade 3), and a CT scan showed grade 1 ILD (Figure 3a). One week later, respiratory disorders and hypoxia were observed, and a CT scan was conducted which demonstrated the progression of ILD (grade 3) (Figure 3b). The patient was diagnosed with EV + P‐induced ILD and was emergently admitted. Upon consultation with the respiratory medicine department, prednisolone at a dose of 50 mg/day was immediately initiated. Despite treatment, on Day 3, oxygenation and pneumonia worsened, prompting initiation of steroid pulse therapy with solumedrol (1000 mg/day). On Day 5, detailed blood examination revealed that the anti‐MDA5 antibody was positive. The patient was diagnosed with dermatomyositis (DM) and administered cyclophosphamide pulse therapy (500 mg/day) and tacrolimus (4 mg/day). However, respiratory distress continued to worsen, and the patient died on day 19 of hospitalization.

## 3 Discussion

We have reported a case of rapidly progressive anti‐MDA5 antibody‐positive ILD induced by EV + P in a patient with advanced UC. To our best knowledge, only two case reports have described ICIs‐induced anti‐MDA5 antibody‐positive ILD in patients with lung cancer or malignant pleural mesothelioma. Thus, this is the first case of anti‐MDA5 antibody‐positive ILD which developed after an ICI‐containing regimen for UC.

IrAEs are thought to be induced by excessive activation of the immune system caused by ICIs [7]. Their clinical manifestations vary widely, most commonly affecting the skin, gastrointestinal, endocrine, and respiratory systems [3]. Notably, the spectrum of fatal irAEs was reported to be caused by pneumonia (35%), hepatitis (22%), and neurotoxicity (15%) [4]. In the present case, the patient had an inherent predisposition to autoimmune disease, DM. Therefore, pembrolizumab, rather than EV, might be associated with the ILD development through immune system activation. Nevertheless, the potential contribution of EV to ILD development should also be considered, as previously reported [8].

Anti‐MDA5 antibody positivity is a diagnostic criterion for DM. In this case, the patient did not present with specific symptoms related to DM, suggesting amyopathic DM (ADM). Baseline creatine kinase levels were not elevated (37 IU/L), and no muscle‐related symptoms such as muscle pain, numbness, or weakness were present. ADM is a distinct subtype characterized by dermatological manifestations of DM without signs of myositis or abnormal laboratory findings, including serologic enzyme levels, electromyography, and muscle biopsy [9]. However, in this case, these specific diagnostic examinations were not performed because of the patient's rapid clinical deterioration and decline in general condition, which may have limited the ability to establish a precise diagnosis of ADM.

Anti‐MDA5 antibody is also associated with rapid progression and aggressiveness of ILD [10]. However, the clinical data on anti‐MDA5 antibody‐positive ILD in patients managed with ICIs are limited. Pan et al. [11] reported the development of anti‐MDA5 antibody‐positive ILD in a patient receiving camrelizumab for lung adenocarcinoma. After the administration of immunosuppressants, the anti‐MDA5 antibody turned negative. However, hypoxia and dyspnea did not improve, and the patient died 2 months after treatment. Kato et al. reported that a patient with malignant mesothelioma rapidly progressed to ILD with anti‐MDA5 antibody 2 weeks after nivolumab administration. Despite initiation of immunosuppressive therapy, the respiratory condition deteriorated owing to the expansion of a fungal empyema and interstitial infiltration, and the patient died [12].

DM increases the risk of cancer development [13, 14], and 12% of patients with DM are at risk of cancer, including bladder cancer [15]. Therefore, it may be reasonable that this patient had advanced bladder cancer together with hidden DM (and anti‐MDA5 antibody positivity).

However, it was difficult to predict this before the initiation of EV + P. In this case, the krebs von den lungen‐6 (KL‐6) level prior to the EV + P initiation was slightly elevated. KL‐6 often increases for nonspecific reasons, such as inflammation or cachexia. Besides, this patient had neither respiratory symptoms nor radiographic abnormality in the lungs. Therefore, we initially considered that the elevation of KL‐6 was not related to an increased risk of ILD induced by EV + P. Nevertheless, owing to the widespread use of ICIs, early screening for anti‐MDA5 antibodies should be considered in patients who develop aggressive ILD during ICI‐containing systemic therapy. Early diagnosis through such screening can provide accurate risk management of this disease and help guide decisions regarding the need for more intensive treatment.

## 4 Conclusion

We report a patient with advanced bladder cancer who developed fatal ILD with anti‐MDA5 antibody after the initiation of first‐line EV + P. This case report emphasizes the importance of immediate screening for anti‐MDA5 antibody in patients who develop rapidly progressive ILD during ICI‐containing systemic therapy for early and optimal treatment.

## Ethics Statement

The authors have nothing to report.

## Consent

The authors have nothing to report.

## Conflicts of Interest

The authors declare no conflicts of interest.

## Data Availability

The authors have nothing to report.
